# Intravenous Irons: From Basic Science to Clinical Practice

**DOI:** 10.3390/ph11030082

**Published:** 2018-08-27

**Authors:** Sunil Bhandari, Dora I. A. Pereira, Helen F. Chappell, Hal Drakesmith

**Affiliations:** 1 Hull and East Yorkshire Hospitals NHS Trust and Hull York Medical School, Hull HU3 2JZ, UK; Sunil.Bhandari@hey.nhs.uk; 2 Department of Pathology, University of Cambridge, Cambridge CB2 1QP, UK; diap2@cam.ac.uk; 3 MRC Unit The Gambia at the London School of Hygiene & Tropical Medicine, Banjul, Gambia; 4 School of Food Science and Nutrition, University of Leeds, Leeds LS2 9JT, UK; H.F.Chappell@leeds.ac.uk; 5 MRC Human Immunology Unit, Weatherall Institute of Molecular Medicine, University of Oxford, Headington, Oxford OX3 9DS, UK; 6 Haematology Theme Oxford Biomedical Research Centre, Oxford OX3 9DS, UK

**Keywords:** adverse event profile, anaemia, bioengineering, labile iron, intravenous iron, iron-carbohydrate complex, iron processing

## Abstract

Iron is an essential trace mineral necessary for life, and iron deficiency anaemia (IDA) is one of the most common haematological problems worldwide, affecting a sixth of the global population. Principally linked to poverty, malnutrition and infection in developing countries, in Western countries the pathophysiology of IDA is primarily linked to blood loss, malabsorption and chronic disease. Oral iron replacement therapy is a simple, inexpensive treatment, but is limited by gastrointestinal side effects that are not inconsequential to some patients and are of minimal efficacy in others. Third generation intravenous (IV) iron therapies allow rapid and complete replacement dosing without the toxicity issues inherent with older iron preparations. Their characteristic, strongly-bound iron-carbohydrate complexes exist as colloidal suspensions of iron oxide nanoparticles with a polynuclear Fe(III)-oxyhydroxide/oxide core surrounded by a carbohydrate ligand. The physicochemical differences between the IV irons include mineral composition, crystalline structure, conformation, size and molecular weight, but the most important difference is the carbohydrate ligand, which influences complex stability, iron release and immunogenicity, and which is a unique feature of each drug. Recent studies have highlighted different adverse event profiles associated with third-generation IV irons that reflect their different structures. The increasing clinical evidence base has allayed safety concerns linked to older IV irons and widened their clinical use. This review considers the properties of the different IV irons, and how differences might impact current and future clinical practice.

## 1. Introduction

Iron, the most abundant element on earth, accounting for 35% of the earth’s mass, is an essential trace mineral necessary for a myriad of metabolic reactions in the body. These include a role in catalytic enzymes and proteins for DNA synthesis, transport of oxygen in haemoglobin and myoglobin, mitochondrial cell respiration, oxidative phosphorylation and adenosine triphosphate (ATP) formation in the tricarboxylic acid cycle [1,2,3]. The human body contains 3–5 g iron and is essential for life, but iron acquisition and assimilation in humans is challenging as oxidised iron is poorly soluble at neutral pH, and within the body, “free” iron is toxic through the promotion of reactive oxygen species. Thus, multiple, complex systems have evolved in man to manage and retain iron, yet iron deficiency and iron deficiency anaemia (IDA) are common problems [4]. Oral iron therapy is a simple, inexpensive treatment, but is limited by gastrointestinal side effects that are not inconsequential to some patients and are of minimal efficacy in others [5]. Advances in our understanding of the pathophysiology of iron metabolism and the development of new pharmaceutical technologies have led to a better understanding of the need for and development of intravenous (IV) formulations for iron replacement when oral iron preparations are not efficacious or cannot be used. This review considers the properties of the different IV irons, and how differences might impact current and future clinical practice.

## 2. Iron Deficiency Anaemia

Iron deficiency is the most common nutritional deficiency worldwide. IDA is one of the most common haematological problems, and the most important cause of a microcytic, hypochromic anaemia. Globally, 1.24 billion people are affected by IDA, which corresponds to about a sixth of the global population [4]. The Global Burden of Disease project highlighted the significant public health importance of IDA, with around 35,000,000 disability-adjusted life years globally attributable to IDA, ranking it fourth in the top leading causes of disability [4]. It is associated with multiple disease states, including chronic kidney disease (CKD), inflammatory bowel disease (IBD) and congestive heart failure and contributes to loss of wellbeing and poor outcomes for patients [6,7]. From a health system perspective, identification and correction of perioperative IDA reduces rates of transfusion and mortality rates, as well as length of hospital stay [8,9]. Regardless of whether IDA is symptomatic, all patients should be treated—both by addressing the underlying cause of the iron deficiency and through adequate replenishment of iron stores. More recently, emerging data also suggest benefit in the treatment of non-anaemic iron deficiency (NAID) with replacement iron [10,11].

### 2.1. Pathophysiology of IDA

Principally linked to poverty, malnutrition and infection in developing countries, in western countries the pathophysiology of IDA is primarily linked to blood loss, malabsorption and chronic disease (Table 1) [7]. Chronic blood loss occurs in a range of conditions that include peptic ulcer disease, inflammatory bowel diseases, regular haemodialysis therapy, occult intestinal cancer and heavy menstrual bleeding. Impaired iron absorption is often apparent after gastrectomy and in inflammatory bowel diseases, and chronic diseases such as CKD are characterised by impaired erythropoiesis through iron restriction. Loss of iron in the urine can occur through rare forms of intravascular haemolysis, and IDA can be drug-related (proton-pump inhibitors, leading to impaired absorption due to increased gastric pH) and genetic (iron-refractory iron-deficiency anaemia, IRIDA) [7]. 

**Figure 1:**
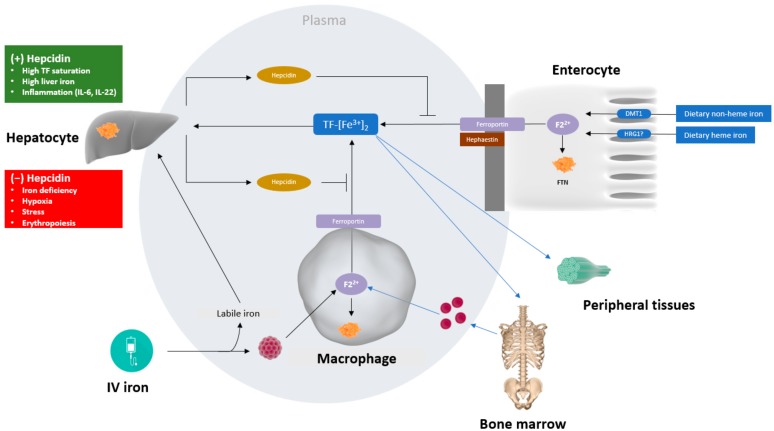
A simplified overview of iron metabolism. The major flow of iron in the body is via transferrin (TF), which transports iron from iron absorption in the enterocytes in the duodenum, the recycling of iron from senescent erythrocytes within macrophages of the reticuloendothelial system, and the mobilisation of iron storage within ferritin (FTN), that mainly resides within macrophages and hepatocytes. Iron export to plasma is mediated via the sole iron exporter, ferroportin and is controlled by its ligand, hepcidin, which is secreted into blood plasma mainly by hepatocytes. Hepcidin binds to ferroportin and controls ferroportin concentration through promoting its endocytosis. Almost all plasma iron is bound to TF, but after infusion of iron supplements, labile (unbound) iron may appear in plasma. The molecular structure of third generation IV irons confers stability on the iron complex, with the intention of limiting the amount of labile iron entering the plasma after infusion and ensuring a controlled release of iron from the complex once taken up by macrophages. How the different IV iron complexes are handled in the macrophage and the basis for their different solubility is not well characterised, but will be dependent not only on pH, but the low molecular weight molecules that are present in the lysosome and their different iron-binding affinities and on macrophage polarisation/differentiation. Iron released from the iron–carbohydrate complex is either stored as ferritin or transported out of the macrophage and bound to TF.

IRIDA is caused by mutations/polymorphisms in the gene TMPRSS6 (transmembrane protease, serine 6) [12]. This anaemia offers useful insights into the normal iron-control mechanisms in humans. The consequence of TMPRSS6 loss of function mutations is constitutively high levels of the iron-regulatory hormone hepcidin, a 25-amino acid peptide that together with the sole iron exporter in the body, ferroportin, control iron homeostasis (Figure 1). High levels of hepcidin result in ferroportin loss from cell membranes and cessation of iron export to plasma. This explains the inability to absorb intestinal iron in IRIDA cases and thus the muted response to treatment with oral iron preparations [13]. This can be partly overcome if sufficiently high quantities of oral iron are administered as demonstrated in studies of the phosphate binder ferric citrate [14].

Hepcidin and iron regulatory proteins (IRPs) are essential for maintaining iron homeostasis [15]. Hepcidin production from the liver is increased in the presence of inflammation, in particular in the presence of the inflammatory cytokine interleukin-6 and is an underlying mechanism of IDA associated with chronic disease [16]. Chronic inflammation, for example related to CKD, increases hepcidin production, in turn inhibiting both the uptake of dietary iron and the mobilization of stored iron from the reticuloendothelial system to circulating transferrin [17]. This restricts the availability of iron for erythropoiesis, which is often superimposed on underlying true iron deficiency and may, therefore, be termed functional iron deficiency as there are sufficient iron stores but an inability to access them due to elevated hepcidin [18,19].

### 2.2. Iron Supplementation Strategies

Historically, oral iron replacement therapy to treat IDA in the form of iron salts dates to the 17th century. Oral ferrous salts are the most commonly prescribed iron replacement therapy, reflecting their efficacy and simplicity of dosing [6,7]. However, long-term treatment of up to 6 months is usually required to adequately replete iron stores, and gastrointestinal side-effects that include nausea and pain are common. A meta-analysis of 43 randomised, controlled trials of 6831 patients reported gastrointestinal side-effects in up to 75% of patients [5]. These side effects can be underestimated regarding their impact on patients and adherence can be challenging [20].

For those who do not respond to oral iron, understanding the hepcidin–ferroportin axis has provided mechanistic insights into relative “iron resistance”. This points to a potential role for hepcidin measurement in clinical practice, with a view to identifying patients most likely or least likely to respond to oral iron therapy. Also, recent studies of NAID have shown that alternate-day dosing of oral iron might optimise iron absorption, as the hepcidin levels fall in the alternate days without iron consumption [10,11].

There is ongoing research interest in the development of original oral irons; in particular, the utilisation of nanotechnology to create novel oral iron nanoformulations [21,22,23,24,25]. One of the strategies proposed is to use the iron core of ferritin, the primary iron-storage protein in cells, as a model. Ferritin has evolved to serve as a highly efficient iron storage protein that is conserved across eukaryotes. Ferritin is composed of an iron oxide nanocore contained within a globular heteropolymeric protein, from which iron release is restricted and controlled [26]. Recently, a nanoparticulate mimetic of the ferritin core was proposed as a potentially side effect-free form of supplemental iron [27] and a paediatric trial is ongoing (NCT02941081). Other trials are on-going with many different oral iron formulations developed to improve absorption and/or tolerability [14,23,28,29,30].

The first parenteral iron preparations to be used clinically in the early 20th century were colloidal ferric hydroxide preparations, but toxicity linked to the release of large amounts of labile (“free”) iron limited their use. This prompted the development of preparations composed of an iron core and carbohydrate shell that prevented rapid release of the elemental iron [13,31]. The introduction of IV iron saccharide in 1947 and high-molecular-weight dextran (HMWD) iron in 1954 signalled a major change in perception of IV iron supplementation due to their efficacy and relative safety. Nonetheless, cases of severe hypersensitivity reactions, in particular the well-documented dextran-induced anaphylactic reactions, led to extreme caution within the medical community regarding the use of these IV irons [13,31].

In the 1990s, two new formulations—iron gluconate and iron sucrose—were developed that used non-dextran carbohydrates complexed with the iron core and these were associated with markedly fewer severe adverse events (SAE). It was shown that patients previously sensitive to HMWD were unlikely to be sensitised to these newer irons [13,31,32]. Development of new pharmaceutical technologies allowed the development of third generation IV irons in an attempt to circumvent the toxicity issues inherent with earlier preparations and the posology limitations of iron sucrose products. In the last ten years, three third-generation IV iron compounds were licensed for the treatment of IDA [13,31]. Two are currently approved for use in Europe—ferric carboxymaltose and iron isomaltoside 1000—and one in the United States—ferumoxytol [33].

## 3. Bioengineering and Metabolism of IV Iron

**Figure 2:**
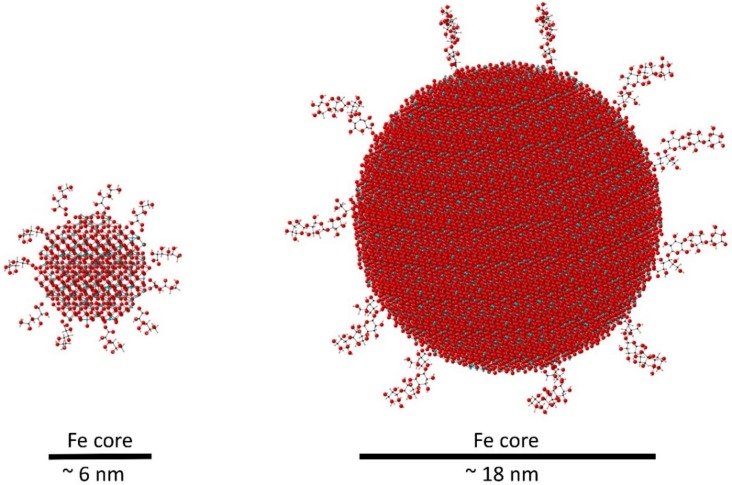
Schematic illustration of the relative size and composition of iron gluconate (left, low molecular weight, 37,500 Da) and iron carboxymaltose (right, high molecular weight, 150,000 Da). The models show iron-oxide cores, based on the neutron diffraction-derived structure of ferrihydrite [40], surrounded by the relevant organic ligands. Oxygen is shown in red, hydrogen in pink, carbon in black and iron in blue.

IV iron preparations are bioengineered as iron-carbohydrate complexes to deliver high doses of iron in a stable, non-toxic form [34] and consist of colloidal suspensions of iron oxide nanoparticles with a polynuclear Fe(III)-oxyhydroxide/oxide core surrounded by a carbohydrate ligand [13,31,35,36,37,38,39] (Table 2). In essence, IV irons behave as prodrugs, retaining ionic iron until the iron–carbohydrate complex is metabolised [35,36]. The physicochemical differences between the IV irons include mineral composition, crystalline structure, conformation, size and molecular weight, but the key point of difference between IV iron products is the carbohydrate ligand, which influences complex stability, iron release and immunogenicity, and is a unique feature of each drug [13,31,34,35,36,37,38] (Table 3). Schematic models of a high molecular weight iron–carbohydrate complex (iron carboxymaltose) and a low molecular weight complex (iron gluconate) are compared in Figure 2, which illustrates that these are two different particles both in terms of overall size but also, and importantly, in terms of number of iron atoms and how accessible these are to undergo chemical reactions. Iron carboxymaltose contains around 110,000 iron atoms bound to 180,000 oxygen atoms in a dense structure with an approximate core diameter of 18 nm. This makes it more difficult to break down once inside the cells, since the iron atoms are less accessible to chelators or redox reactions, whereas iron gluconate is smaller with a 6 nm diameter, is less compact and has fewer iron atoms (around 4200 iron atoms bound to 7000 oxygen atoms) and, therefore, the kinetics of iron release from iron gluconate is faster than that of iron release from iron carboxymaltose, meaning that iron gluconate is more labile than iron carboxymaltose.

Upon IV infusion, the volume of distribution of IV irons corresponds roughly to the plasma volume. IV irons are processed in macrophages and release functional iron from the carbohydrate ligand [35,36]. The iron complex is endocytosed by macrophages within the reticuloendothelial system, mainly in the liver, spleen and bone marrow, but the precise mechanism of recognition and internalization is not fully defined [41]. Endosome–lysosome fusion creates an acidic endolysosome and, combined with endogenous iron-binding due to citric acid and other iron-complexing agents present in the lysosomes, drives iron release from the iron–carbohydrate complex [42]. This may be distinct from the mechanism of iron release from ferritin. Early in-vitro stability analyses suggest that the low pH of the endolysosome, the type and concentration of low-molecular-weight iron-ligands present in the endolysosome and the stabilities of the different IV iron-carbohydrate structures, are all important for iron liberation [42,43]. However, the precise mechanism of iron liberation is incompletely understood. This is an area of current interest—IV iron metabolism is likely to vary depending on the type and differentiation state of the macrophage processing the iron [44,45], and iron itself can alter macrophage polarisation [46], so that the different characteristics of each iron-carbohydrate complex may potentially affect macrophage function. The latter may well have relevance for the regulatory authorities when defining the extent of similarity between iron-formulations of the same class.

Iron is subsequently transported into the labile iron pool in the macrophage cytoplasm, where it can be stored, or exported into the plasma by ferroportin. The mechanism by which intracellular iron is delivered to ferroportin for export is not well characterised [47]. Upon export, iron is immediately oxidised by ferroxidases, and sequestered by plasma transferrin for transport to erythroid precursor cells for incorporation into haemoglobin or to other iron-requiring cells, or for liver storage in the form of ferritin [35,36,37] (Figure 1).

**Figure 3:**
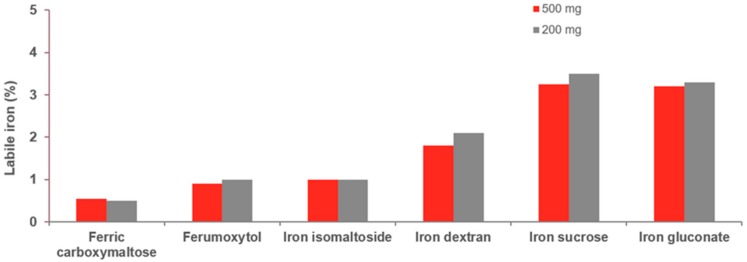
In vivo comparison of labile iron potential of IV iron preparations. Results of the determination of detectable labile iron in human serum as a percentage of total dose given (200 mg and 500 mg), using the Ferrozine^®^-method (adapted from Jahn et al., 2011 [32]).

The stability of the iron–carbohydrate complex influences the amount of labile iron that is present in the formulation [32,37]. Strongly-bound iron–carbohydrate complexes characterise the third-generation IV irons. These stable, robust complexes bind iron tightly and do not release large amounts of labile, non-transferrin-bound iron (NTBI) into the blood before macrophage uptake. As a result, the risk of infusion reactions caused by labile iron is diminished and are clinically well-tolerated even at high doses [13,31,32,35,37,39,48,49]. This allows rapid, high-dose infusion of doses of 1000–1500 mg, thus offering the potential for complete iron replacement in 15–60 min (Table 2), although processing and distribution of the iron will obviously take longer. Comprehensive biochemical quantification of the different IV irons preparations confirms differences in complex stability and labile iron release (Figure 3) [32].

Compared with third-generation irons, the lower molecular weight iron sucrose complexes have lower complex stability, are termed semi-robust, moderately strong, and release larger amounts of NTBI into the blood [37]. As a result, maximal single doses are significantly lower with longer administration times [33,37]. Ferric gluconate preparations contain variable amounts of low-molecular-weight components (<18,000 Daltons), are characteristically labile and weak, and generate larger amounts of labile NTBI [37]. Labile iron also has the potential for formation of highly reactive free radicals causing oxidative stress [50]. Under normal physiological conditions, iron in the body exists in a non-redox-active form, i.e., it is not able to repetitively complete coupled reduction and oxidation, as in ferritin and transferrin. However, should iron infusions lead to complete or near-complete saturation of transferrin, the subsequent high levels of labile, redox-active iron, particularly with high-dose infusion, may contribute to formation of reactive oxygen species and reactive nitrogen species in an uncontrolled way. These entities cause oxidative and/or nitrosative stress that upsets normal cellular signalling mechanisms and has been shown to be involved in many diseases, including heart failure, and Alzheimer’s disease, Friedreich’s ataxia and Parkinson’s disease [35,39].

A further consideration is the risk of dextran-related immune reactions and clinical hypersensitivity reactions more generally. Dextran, given intravenously, can result in IgG-mediated anaphylaxis [34]. Early IV formulations of high-molecular-weight iron dextran caused rare but serious allergic reactions that led to anaphylaxis [13,31]. The formulation of iron dextran (lower molecular weight) currently available is associated with markedly lower rates of adverse events [51,52,53,54]. For third-generation IV iron preparations, the carbohydrate component of the iron-carbohydrate complex is responsible for immune recognition and eliciting hypersensitivity reactions. It is now thought that a complement activation-related pseudo-allergy is likely a more common pathogenetic mechanism in acute reactions to currently licensed IV iron than a response that is immunological and IgE-mediated [55]. The response is likely triggered by iron nanoparticles and influenced by the unique carbohydrate-iron complex of each IV iron.

Thus, the structure and physiochemical characteristics of IV irons have implications for therapy, impacting the maximum amount of iron that can be infused, the rate of infusion, the risk of minor infusion reactions, the risk of immune-mediated hypersensitivity reactions, and wider negative effects linked to the toxicity of labile iron.

### Regulatory View of IV Iron Formulations and Bioequivalence

The complexity of the IV iron formulations underlies the ongoing issue regarding approval of generic copies of non-biologic complex drugs. The authorisation process for generic pharmaceuticals is currently based on pharmaceutical equivalence which encompasses demonstration of ‘sameness’ of the active pharmaceutical ingredient and bioequivalence to the listed reference product without the need for efficacy studies and establishing a safety profile. With the availability of iron sucrose similars in the European Union, differences in clinical efficacy and safety profiles to the originator drug have been reported [56,57,58,59,60,61,62,63].

With this background, the regulators have taken notice of the complexity of IV iron formulations. Both the Food and Drug Administration (FDA) and European Medicines Agency (EMA) acknowledge that IV iron–carbohydrate nanoparticle preparations cannot be authorised by this well-established generic approval paradigm for small molecules. The EMA and FDA have reflected on the data requirements to define similarity and the possibility of interchanging or switching between different IV iron formulations; these include stepwise in vitro, non-clinical and clinical testing as a prerequisite [64,65,66]. The EMA has concluded that the stability of IV iron-carbohydrate complexes and the physicochemical properties of both the iron and the carbohydrate impact the quality attributes of the different drugs, which have the potential to influence both safety and efficacy [66]. This position draws into question the view that IV irons are substitutable and interchangeable and suggests caution should be exercised when switching between IV irons, with appropriate efficacy and safety monitoring put in place.

## 4. Clinical Use of IV Iron

The prescribing of IV irons across a broad range of indications reflects the breadth of conditions that manifest IDA. IV iron use has dramatically increased, and the broad and increasing number of conditions for which IV iron has been investigated is at least in part supported by their safety profile. An extensive meta-analysis of >10,000 patients derived from 103 clinical trials offers important insights into the overall safety profile and allows comparison between IV and oral iron [67]. IV iron was not associated with an increase in serious AEs (SAE) when compared to oral iron and placebo (RR, 1.04; 95% CI, 0.93–1.14). SAE were rare, estimated to occur in 1:200,000 doses with no fatal or anaphylactic reactions reported [67]. Although the study confirmed that minor infusion reactions do occur, the frequency of these adverse events must be considered in the context of the use of blood transfusions. In many cases, transfusions are the only alternative to IV iron to correct IDA when oral iron is ineffective or not tolerated or in acute situations when there is not enough time for oral iron to replenish iron levels.

A limitation of the meta-analysis is the short-term nature of randomised clinical trials (RCTs), with long-term adverse events not being tracked (67). For conditions such as CKD that are dependent on ongoing long-term dialysis and frequent iron infusions, there are some concerns about the long-term safety outcomes. This concern in part relates to the potential for oxidative stress linked to IV iron use, especially in the context of the ongoing chronic inflammatory processes characteristic of CKD. A large, clinical study (proactive IV iron therapy in haemodialysis patients, PIVOTAL, EudraCT number 2013-002267-25) has recently been completed and will report major adverse outcomes including major cardiovascular events in CKD patients [68].

There is extensive clinical trial evidence supporting the efficacy of IV iron preparations in patients with both non-dialysis-dependent and end-stage CKD [69,70,71,72,73,74,75,76,77,78,79,80,81,82,83,84,85,86,87,88,89,90,91,92,93,94,95,96,97,98,99] and patients with CHF [100,101,102,103,104,105,106,107,108,109,110], IBD [28,111,112,113,114,115,116,117,118,119,120], women’s health (including abnormal uterine bleeding [121], peri and postpartum IDA [122,123,124,125], and prevention in pregnancy [126], and in various cancers [127,128,129,130,131], and in the perioperative management of anaemia [132]. Several observational cohort studies further support the efficacy and safety of IV iron in real-world clinical practice for IDA associated with CKD [133,134,135,136,137,138,139,140,141,142] (including dialysis patients hospitalised for infection [139]), IBD [143,144,145], pregnancy [146] and cancer [147,148].

Multiple clinical guidelines relating to the management of iron deficiency in various healthcare fields including CKD, CHF, IBD, cancer and pregnancy note the benefit of IV iron as a treatment option for CKD for whom oral iron is not an option (lack of response, non-compliance, or intolerance) and for those patients with severe iron deficiency with the need for rapid iron replenishment [149,150]. As would be expected, the clinical evidence base underpinning these guidelines is strong. The 2018 European Society for Medical Oncology (ESMO) clinical practice guidelines for the management of anaemia and iron deficiency in patients with cancer reflect the most recent clinical data and strongly recommend IV iron in confirmed iron deficiency [151]. For chemotherapy-associated anaemia, IV iron makes an important contribution to improved response to erythropoiesis stimulating agents. In dialysis associated with CKD, IV iron is standard care practice [152].

### Evolving Evidence Base Identifies Differences between Third-Generation IV Irons

Generally, reviews and meta-analyses provide valuable pooled results reporting the overall efficacy and safety profile of the class. Direct comparison between different irons are few, but with the increasing evidence pool and broadening of diseases assessed for IDA correction, some differences between irons are beginning to become apparent that may impact clinical practice. 

In heart failure, iron deficiency is linked to disease severity and is only partly related to anaemia and is recognised as a marker of poor prognosis, independent of other prognostic factors [153,154,155]. IV iron improves patient well-being and New York Heart Association (NYHA) functional class, as reported in the FAIR-HF trial (Ferinject Assessment in Patients with Iron Deficiency and Chronic Heart Failure) in both anaemic and non-anaemic patients and exercise capacity over 24 weeks in the CONFIRM-HF trial (Ferric CarboxymaltOse evaluatioN on perFormance in patients with IRon deficiency in coMbination with chronic Heart Failure) [107,156]. In light of the evidence derived from trials of ferric carboxymaltose, the European Society of Cardiology guidelines for heart failure management makes a specific recommendation that ferric carboxymaltose should be considered in symptomatic patients to alleviate HF symptoms and improve exercise capacity and quality of life [150]. Whether other third generation irons have a similar impact is yet to be confirmed but is currently being investigated with results expected in 2021 (NCT02642562) [157].

Iron deficiency is highly prevalent in IBD, occurring in more than two-thirds of patients with Crohn’s disease and ulcerative colitis [158,159]. Inflammatory bowel disease is perhaps a unique disease process with respect to IDA because of the multiple pathogenic mechanisms involved in its pathophysiology. Chronically inflamed intestinal mucosa with blood loss and micronutrient deficiency (iron and B12) are the primary mechanisms underlying the development of anaemia in IBD, with chronic inflammation, haemolysis, and medication-induced myelosuppression also thought to have roles [160]. A Bayesian network meta-analysis assessed the comparative efficacy and safety of different IV irons in patients with IBD and anaemia. This meta-analysis included five trials involving a relatively small number of patients (*n* = 1143) treated with IV iron (IS, ferric carboxymaltose, ferumoxytol, low-molecular-weight iron dextran, ferric gluconate, iron isomaltoside). Based on response rate, defined as Hb normalisation and/or increase by ≥2 g/dL, ferric carboxymaltose alone was shown to have superior efficacy than oral iron (OR = 1.9, 95% CI: 1.1; 3.2) [161].

From a safety perspective, the two main side effects associated with IV iron are hypophosphataemia, apparent soon after infusion and lasting up to 2 weeks, and hypersensitivity reactions, as previously noted. The occurrence of hypophosphataemia has been investigated further. The underlying cause of the phenomenon is related to the expression of FGF-23, a hormone derived from osteocytes, with the ultimate consequence of renal phosphate excretion. A head-to-head retrospective analysis of 81 patients who received ferric carboxymaltose or iron isomaltoside identified hypophosphataemia almost exclusively in the ferric carboxymaltose-treated patients and caused by increased concentrations of FGF-23 [162]. A more recent study, the FIRM study in 1997 participants also demonstrated this difference in effect on phosphate when ferric carboxymaltose was compared with ferumoxytol [163]. This side effect highlights a subtle but potentially important different pharmacodynamic action of these two third generation IV irons. More data on hypophosphataemia is accruing; it is currently thought to be generally mild and usually without clinical consequence, but rare cases with clinical sequalae are apparent and this issue must be kept in consideration. The risk may be increased in patients with other disturbances of phosphate metabolism, such as hyperparathyroidism. A Danish retrospective analysis of 231 outpatients treated with IV iron infusions over a 2.5-year period, during which ferric carboxymaltose was switched to iron isomaltoside and back to ferric carboxymaltose in a stable cohort of patients largely with IBD [164], showed hypophosphataemia events were markedly more frequent in patients treated with ferric carboxymaltose than iron isomaltoside (64 vs. 9, *p* < 0.01). The study also reported that significantly more patients experienced hypersensitivity reactions with iron isomaltoside than ferric carboxymaltose (2.5% vs. 10.7%, *p* < 0.01), with no hypersensitivity crossover between the two drugs apparent. With the same amount of iron delivered with both drugs, this study suggests the differences in side effect profile relate to the different structures and properties of the two different IV iron drugs. More recent reviews have reassured that at least in patients with renal disease, the prevalence of serious adverse reactions is relatively low [49,67,165,166].

## 5. Conclusions

IDA is a clinically important complication associated in particular with chronic inflammatory conditions, infection and other disease states and leads to chronic fatigue, reduced quality of life (QOL), increased risk of complications and increased mortality. Treatment of iron deficiency falls into two main categories, oral and IV iron formulations [167]. Clinical practice guidelines identify the benefits of IV iron preparations as a treatment option for patients with IDA who lack a response to, are non-compliant with, or are intolerant of oral iron treatment, as well as those who have severe iron deficiency and require rapid replenishment of available iron and Hb levels. IV iron has an important role in management of IDA perioperatively, particularly for emergency surgery, and there is now a robust clinical trial evidence base supporting the efficacy and safety of IV iron preparations in CKD, CHF, IBD, women’s health and cancer.

The third generation of IV irons are characterised by unique carbohydrate ligands forming strongly bound iron–carbohydrate complexes. Their complexity is reflected in the guidance from regulators that generic formulations cannot be authorised by the generic approval process and goes against the view that IV irons are substitutable and interchangeable. The risk of infusion reactions is diminished compared with previous IV iron formulations, and these drugs are clinically well-tolerated at high doses to allow rapid, high-dose infusion that offers the potential for complete iron repletion in 15–60 min. As the evidence base and range of indications for which IV iron treatment is used expands, it is valuable to define the differences between these agents, in particular adverse event profiles, and reflect on how this might influence both the choice of iron and the decision to switch between IV irons in clinical practice. 

## Figures and Tables

**Table 1:** Main causes of iron deficiency in the western world [7].

Cause	Details
Insufficient uptake	Malnutrition or diet-related (low-iron, vegetarian, vegan)
Increased physiological demand	Rapid growth during infancy/adolescence, menstrual blood loss, pregnancy (2nd/3rd trimesters)
Chronic blood loss	Trauma, surgery, delivery, heavy menstrual bleeding
Chronic disease	Kidney disease, heart failure, inflammatory bowel disease, gastritis, peptic ulcer, intestinal cancer and benign tumours
Drug-related	Glucocorticoids, salicylates, non-steroidal anti-inflammatory drugs, proton-pump inhibitors, H_2_-receptor antagonists, drug-induced haemolytic anaemia
Genetic	Iron-refractory iron-deficiency anaemia, thalassaemia and sickle cell anaemia

**Table 2:** Clinical characteristics of currently available IV irons [13,33,39].

	Ferumoxytol	Iron Carboxymaltose	Iron Isomaltoside 1000	Low Molecular Weight Iron Dextran	Iron Sucrose	Iron Gluconate
**Brand name**	Feraheme^®^	Ferinject^®^	Monofer^®^	Cosmofer^®^	Venofer^®^	Ferlixit^®^
**Maximum single dose**	510 mg	1000 mg	20 mg/kg	20 mg/kg	200 mg	125 mg
**Minimum administration time (minutes)**	15	15	15	60	30	30–60
**Replacement dose possible in a single infusion**	No	Yes	Yes	Yes	No	No

**Table 3:** Comparison of physicochemical characteristics and pharmacokinetics of IV irons [37,38,39].

	Ferumoxytol	Iron Carboxymaltose	Iron Isomaltoside 1000	Low Molecular Weight Iron Dextran	Iron Sucrose	Iron Gluconate
**Molecular weight (Da)**	185,000	150,000	150,000	103,000	43,000	37,500
**Carbohydrate ligand**	Polyglucose sorbitol carboxymethylether	Carboxymaltose	Isomaltoside	Dextran polysaccharide	Sucrose	Gluconate, loosely associated sucrose
**Relative stability of iron carbohydrate complex**	High	High	High	High	Medium	Low
**Reactivity with transferrin**	Low	Low	Low	Low	Medium	High
**Relative labile iron release**	Low	Low	Low	Medium	High	High
**Plasma half-life (hrs)**	~15	7–12	20	5–20	6	~1
